# High-Order Compact Difference Scheme for the Numerical Solution of Time Fractional Heat Equations

**DOI:** 10.1155/2014/642989

**Published:** 2014-02-13

**Authors:** Ibrahim Karatay, Serife R. Bayramoglu

**Affiliations:** Department of Mathematics, Fatih University, 34500 Istanbul, Turkey

## Abstract

A high-order finite difference scheme is proposed for solving time fractional heat equations. The time fractional derivative is described in the Riemann-Liouville sense. In the proposed scheme a new second-order discretization, which is based on Crank-Nicholson method, is applied for the time fractional part and fourth-order accuracy compact approximation is applied for the second-order space derivative. The spectral stability and the Fourier stability analysis of the difference scheme are shown. Finally a detailed numerical analysis, including tables, figures, and error comparison, is given to demonstrate the theoretical results and high accuracy of the proposed scheme.

## 1. Introduction

In the last decades, more and more attention has been placed on the development and research of fractional differential equations, because they can describe many phenomena, physical and chemical processes more accurately than classical integer order differential equations [1–4]. And the finite difference method is an efficient tool for solving fractional partial differential equations.

There are many different discretizations in time variable equipped with the compact difference scheme in spatial variable. The approximations given in [4–9] are of the order *O*(*τ* ^*μ*^ + *h* ^4^), where 1 ≤ *μ* < 2. Here, we propose a method for the time fractional differential heat equations with the accuracy of order *O*(*τ* ^2^ + *h* ^4^).

In this work, we consider the following time fractional heat equation:

(1) $$\begin{array}{c} \frac{\partial_{M}^{\alpha }u\left(t,x\right)}{\partial t^{\alpha }}=\frac{\partial^{2}u\left(t,x\right)}{\partial x^{2}}+f\left(t,x\right),\;0<x<1,\;\;0<t<1,\\ u\left(0,x\right)=r\left(x\right),\;0\leq x\leq 1,\\ u\left(t,0\right)=0,\;u\left(t,1\right)=0,\;0\leq t\leq 1, \end{array}$$

where the term ∂_*M*_^*α*^ *u*(*t*, *x*)/∂*t* ^*α*^ denotes *α*-order modifying Riemann-Liouville fractional derivative [10] given with the following formula:

(2) ∂Mαu(t,x)∂tα ={1Γ(1−α)∂∂t∫0tu(s,x)−u(0,x)(t−s)αds,if  0<α<1,∂∂tu(t,x),if α=1,

where Γ(·) is the gamma function.

**Remark 1**

If *r*(*x*) = 0, then the Riemann-Liouville and the modified Riemann-Liouville fractional derivatives are identical, since the Riemann-Liouville derivative is given by the following formula [11]:

(3) $$\begin{array}{c} \frac{\partial^{\alpha }u\left(t,x\right)}{\partial t^{\alpha }}=\{\begin{array}{cc} \frac{1}{\Gamma \left(1-\alpha \right)}\frac{\partial }{\partial t}\int_{0}^{t}\frac{u\left(s,x\right)}{{\left(t-s\right)}^{\alpha }}ds, & \text{if}\;\;0<\alpha <1,\\ \frac{\partial }{\partial t}u\left(t,x\right), & \text{if}\;\alpha =1. \end{array} \end{array}$$

If *r*(*x*) is nonzero, then there are some problems about the existence of the solutions for the heat equation (1). To rectify the situation two main approaches can be used; the modified Riemann-Liouville fractional derivative can be used [10] or the initial condition should be modified [12]. We chose the first approach in our work.

## 2. Discretization of the Problem

In this section we introduce the basic ideas for the numerical solution of the time fractional heat equation (1) by compact finite difference scheme.

For some positive integers *M* and *N*, the grid sizes in space and time for the finite difference algorithm are defined by *h* = 1/*M* and *τ* = 1/*N*, respectively. The grid points in the space interval [0,1] are the numbers *x*_*j*_ = *jh*, *j* = 0,1, 2,…, *M*, and the grid points in the time interval [0,1] are labeled *t*_*k*_ = *kτ*, *k* = 0,1, 2,…, *N*. The values of the functions *U* and *f* at the grid points are denoted by *U*_*j*_^*k*^ = *U*(*t*_*k*_, *x*_*j*_) and *f*_*j*_^*k*^ = *f*(*t*_*k*_, *x*_*j*_), respectively.

As in the classical Crank-Nicholson difference scheme, we use the approximation [13] to the fractional derivative ∂^*α*^ *U*(*t*, *x*)/∂*t* ^*α*^ at (*t*_*k*+1/2_, *x*_*j*_), and then

(4) ∂αU(tk+1/2,xj)∂tα =∂∂tH(tk+1/2,xj) =H(tk+1,xj)−H(tk,xj)τ+O(τ2) =w0Ujk+1+∑r=1k(wr−wr−1)Ujk+1−r−wkUj0+O(τ2),

where *H*(*t*, *x*) = (1/Γ(1 − *α*))∫_0_^*t*^((*u*(*s*, *x*) − *u*(0, *x*))/(*t*−*s*)^*α*^)*ds*, *w*_0_ = *b*_0_ − *a*_0_, and *w*_*k*_ = *a*_*k*−1_ − *a*_*k*_ + (*k* + 1)*b*_*k*_ − (*k* − 1)*b*_*k*−1_, for 1 ≤ *k* ≤ *N*.

**Definition 2**

Define the average operator *κ* : *ϑ* → *ϑ* as follows:

(5) $$\begin{array}{c} {\left(\kappa g\right)}_{j}=\{\begin{array}{cc} g_{0}, & j=0,\\ \frac{1}{12}\left(g_{j-1}+10g_{j}+g_{j+1}\right), & 1\leq j\leq M-1,\\ g_{M}, & j=M, \end{array} \end{array}$$

where *g* = (*g*_0_, *g*_1_,…, *g*_*M*_) is a grid function and *ϑ* is the space of the grid functions.

**Lemma 3**

Suppose *p*(*x*) ∈ *C* ^6^[0,1], then

(6) κ∂2p(xi)∂x2=1h2[p(xi−1)−2p(xi)+p(xi+1)] +O(h4), 1≤i≤M−1.

**Proof**

We use the Taylor expansion of each term about *x*_*i*_, and then we obtain the following truncation error for any *i*, where 1 ≤ *i* ≤ *M* − 1:

(7) κ∂2p(xi)∂x2−1h2[p(xi−1)−2p(xi)+p(xi+1)] =112[p′′(xi+1)+10p′′(xi)+p′′(xi−1)]  −1h2[p(xi+1)−2p(xi)+p(xi−1)] =h4360∫01[p(6)(xi+sh)+p(6)(xi−sh)]      ×(1−s)3[5−3(1−s)2]ds =h4240p(6)(ξi), ξi∈(xi−1,xi+1).

## 3. Compact Finite Difference Scheme

If we apply the operator *κ* to both side of (1),

(8) $$\begin{array}{c} \kappa \frac{\partial_{M}^{\alpha }u\left(t,x\right)}{\partial t^{\alpha }}=\kappa \frac{\partial^{2}u\left(t,x\right)}{\partial x^{2}}+\kappa f\left(t,x\right),\;0<x<1,\;0<t<1,\\ u\left(0,x\right)=r\left(x\right),\;0\leq x\leq 1,\\ u\left(t,0\right)=0,\;u\left(t,1\right)=0,\;0\leq t\leq 1, \end{array}$$

and use the approximation (4), then we obtain the following difference scheme which is accurate of order *O*(*τ* ^2^ + *h* ^4^);

(9) 112[w0Uj−1k+1+∑r=1k(wr−wr−1)Uj−1k+1−r−wkUj−10]  +1012[w0Ujk+1+∑r=1k(wr−wr−1)Ujk+1−r−wkUj0]  +112[w0Uj+1k+1+∑r=1k(wr−wr−1)Uj+1k+1−r−wkUj+10] =[Uj+1k+1−2Ujk+1+Uj−1k+12h2+Uj+1k−2Ujk+Uj−1k2h2]  +112[f(tk+τ2,xj−1)+10f(tk+τ2,xj)     +f(tk+τ2,xj+1)],            0≤k≤N−1, 1≤j≤M−1,Uj0=r(xj), 1≤j≤M−1,U0k=0, UMk=0, 0≤k≤N.

The difference scheme above can be written in matrix form as follows:

(10) $$\begin{array}{c} AU_{j+1}+BU_{j}+AU_{j-1}=\varphi_{j}, \end{array}$$

where *φ*_*j*_ = [*φ*_*j*_^0^, *φ*_*j*_^1^, *φ*_*j*_^2^,…, *φ*_*j*_^*N*^]^*T*^, *φ*_*j*_^0^ = *r*(*x*_*j*_), *φ*_*j*_^*k*^ = *f*(*t*_*k*+1/2_, *x*_*j*_), 1 ≤ *k* ≤ *N*, 1 ≤ *j* ≤ *M*, and *U*_*j*_ = [*U*_*j*_^0^,*U*_*j*_^1^,*U*_*j*_^2^,…,*U*_*j*_^*N*^]^*T*^.

Here *A*_(*N*+1)×(*N*+1)_ and *B*_(*N*+1)×(*N*+1)_ are the matrices of the form:

(11) $$\begin{array}{c} A=\left[\begin{array}{cccccc} 0 & & & & & \\ \frac{-w_{0}}{12}-\frac{1}{2h^{2}} & \frac{w_{0}}{12}-\frac{1}{2h^{2}} & & & & \\ \frac{-w_{1}}{12} & \frac{w_{1-}w_{0}}{12}-\frac{1}{2h^{2}} & \frac{w_{0}}{12}-\frac{1}{2h^{2}} & & & \\ \frac{-w_{2}}{12} & \frac{w_{2-}w_{1}}{12} & \frac{w_{1-}w_{0}}{12}-\frac{1}{2h^{2}} & \frac{w_{0}}{12}-\frac{1}{2h^{2}} & & \\ & & \ddots & \ddots & \ddots & \\ \frac{-w_{N-1}}{12} & \frac{w_{N-1-}w_{N-2}}{12} & \cdots & \frac{w_{2-}w_{1}}{12} & \frac{w_{1-}w_{0}}{12}-\frac{1}{2h^{2}} & \frac{w_{0}}{12}-\frac{1}{2h^{2}} \end{array}\right],\\ B=\left[\begin{array}{ccccc} 1 & & & & \\ \frac{-10w_{0}}{12}+\frac{1}{h^{2}} & \frac{10w_{0}}{12}+\frac{1}{h^{2}} & & & \\ \frac{-10w_{1}}{12} & \frac{10\left(w_{1}-w_{0}\right)}{12}+\frac{1}{h^{2}} & \frac{10w_{0}}{12}+\frac{1}{h^{2}} & & \\ \frac{-10w_{2}}{12} & \frac{10\left(w_{2}-w_{1}\right)}{12} & \frac{10\left(w_{1}-w_{0}\right)}{12}+\frac{1}{h^{2}} & \frac{10w_{0}}{12}+\frac{1}{h^{2}} & \\ & & \ddots & \ddots & \ddots \\ \frac{-10w_{N-1}}{12} & \frac{10\left(w_{N-1}-w_{N-2}\right)}{12} & \cdots \;\;\frac{10\left(w_{2}-w_{1}\right)}{12} & \frac{10\left(w_{1}-w_{0}\right)}{12}+\frac{1}{h^{2}} & \frac{10w_{0}}{12}+\frac{1}{h^{2}} \end{array}\right]. \end{array}$$

We note that the unspecified entries are zeros at the matrices above.

Using the idea of the modified Gauss-Elimination method, we can convert (10) into the following form:

(12) $$\begin{array}{c} U_{j}=\alpha_{j+1}U_{j+1}+\beta_{j+1},\;j=M-1,\ldots ,2,1,0. \end{array}$$

This way, the two-step form of difference schemes in (10) is transformed to one-step method as in (12).

Now, we need to determine the matrices *α*_*j*+1_ and *β*_*j*+1_ satisfying the last equality. Since *U*_0_ = *α*_1_ *U*_1_ + *β*_1_ = 0, we can select *α*_1_ = *O*_(*N*+1)×(*N*+1)_ and *β*_1_ = *O*_(*N*+1)×1_. Combining the equalities *U*_*j*_ = *α*_*j*+1_ *U*_*j*+1_ + *β*_*j*+1_, and *U*_*j*−1_ = *α*_*j*_ *U*_*j*_ + *β*_*j*_ and the matrix equation (10), we have

(13) (A+Bαj+1+Aαjαj+1)Uj+1  +(Bβj+1+Aαjβj+1+Aβj)=φj.

Then, we write

(14) $$\begin{array}{c} A+B\alpha_{j+1}+A\alpha_{j}\alpha_{j+1}=0,\\ B\beta_{j+1}+A\alpha_{j}\beta_{j+1}+A\beta_{j}=\varphi_{j}, \end{array}$$

where 1 ≤ *j* ≤ *M* − 1.

So, we obtain the following pair of formulas [15]:

(15) $$\begin{array}{c} \alpha_{j+1}=-{\left(B+A\alpha_{j}\right)}^{-1}A,\\ \beta_{j+1}={\left(B+A\alpha_{j}\right)}^{-1}\left(\varphi_{j}-A\beta_{j}\right), \end{array}$$

where 1 ≤ *j* ≤ *M* − 1.

## 4. The Spectral Stability of the Method

The spectral stability analysis is done by using the analysis of the eigenvalues of the iteration matrix *α*_*j*_ (1 ≤ *j* ≤ *M*) of the scheme (12).

Let *ρ*(*A*) denote the spectral radius of a matrix *A*, that is, the maximum of the absolute value of the eigenvalues of the matrix *A*.

We will prove that *ρ*(*α*_*j*_) < 1, (1 ≤ *j* ≤ *M*), by induction

since *α*_1_ is a zero matrix *ρ*(*α*_1_) = 0 < 1.

Moreover, *α*_2_ = −*B* ^−1^ *A*, and *α*_2_ is a lower triangular matrix of the following form:

(16) α2=[0∗−w0/12+1/2h210w0/12+1/h2⋱∗∗−w0/12+1/2h210w0/12+1/h2](N+1)×(N+1).

Therefore *ρ*(*α*_2_) = *ρ*(−*B* ^−1^ *A*) = |(1/2*h* ^2^ − *w*_0_/12)/(1/*h* ^2^ + 10*w*_0_/12)| < |(1/2*h* ^2^ − *w*_0_/12)/(1/2*h* ^2^ + 9*w*_0_/12 + 1/2*h* ^2^ + *w*_0_/12)|<|(1/2*h* ^2^ − *w*_0_/12)/(1/2*h* ^2^ + *w*_0_/12)|.

Since

(17) w0=b0−a0=τ−α(1−α)Γ(1−α)−τ−α(2−α)Γ(1−α)=τ−αΓ(3−α)>0

and 1/*h* ^2^ > 0, we can write *ρ*(*α*_2_) < 1.

Now, assume *ρ*(*α*_*j*_) < 1. After some calculations we find that

(18) αj+1=[−(B+Aαj)−1A](N+1)×(N+1)=[0∗−(w0/12−1/2h2)B2,2+A2,2αj2,2∗∗⋱∗∗−(w0/12−1/2h2)BN+1,N+1+AN+1,N+1αjN+1,N+1],

and we already know that *A*_*i*,*i*_ = *w*_0_/12 − 1/2*h* ^2^, *B*_*i*,*i*_ = 10*w*_0_/12 + 1/*h* ^2^ and *α*_*j*_*i*,*i*__ = *λ*_*j*_, nonzero eigenvalue of *α*_*j*_ for 2 ≤ *i* ≤ *N* + 1.

(19) $$\begin{array}{c} \rho \left(\alpha_{j+1}\right)\;=\left|\frac{1/2h^{2}-w_{0}/12}{1/h^{2}+10w_{0}/12+\left(w_{0}/12-1/2h^{2}\right)\lambda_{i}}\right|. \end{array}$$

If 1/2*h* ^2^ − *w*_0_/12 ≥ 0, then we have two subcases.

(a) If 0 < *λ*_*j*_ < 1,
  (20) ρ(αj)=|1/2h2−w0/121/h2+10w0/12+(w0/12−1/2h2)λj|=1/2h2−w0/121/h2+10w0/12−(1/2h2−w0/12)λj≤1/2h2−w0/121/h2+10w0/12−(1/2h2−w0/12)=1/2h2−w0/121/2h2+11w0/12≤1.
(b) If −1 < *λ*_*j*_ < 0,
  (21) ρ(αj+1)=|1/2h2−w0/121/h2+10w0/12+(w0/12−1/2h2)λj|=1/2h2−w0/121/h2+10w0/12−(1/2h2−w0/12)λj≤1/2h2−w0/121/h2+10w0/12=1/2h2−w0/121/2h2+w0/12+1/2h2+9w0/12≤1/2h2−w0/121/2h2+w0/12<1.

If 1/2*h* ^2^ − *w*_0_/12 < 0, then we have two subcases.

(a) If 0 < *λ*_*j*_ < 1,
  (22) ρ(αj+1)=|1/2h2−w0/121/h2+10w0/12+(w0/12−1/2h2)λj|=−1/2h2+w0/121/h2+10w0/12+(w0/12−1/2h2)λj≤w0/12−1/2h2w0/12+1/2h2+1/2h2+9w0/12=w0/12−1/2h2w0/12+1/2h2≤1.
(b) If −1 < *λ*_*j*_ < 0,
  (23) ρ(αj+1)=|1/2h2−w0/121/h2+10w0/12+(w0/12−1/2h2)λj|=−1/2h2+w0/121/h2+10w0/12+(w0/12−1/2h2)λj≤w0/12−1/2h21/h2+10w0/12−(w0/12−1/2h2)=w0/12−1/2h29w0/12+3/2h2=w0/12−1/2h2w0/12+1/2h2+(8w0/12+1/h2)≤w0/12−1/2h2w0/12+1/2h2<1.

So, we have proved that whenever *ρ*(*α*_*j*_) < 1 then it follows that *ρ*(*α*_*j*+1_) < 1. So *ρ*(*α*_*j*_) < 1 for any *j*, where 1 ≤ *j* ≤ *M*.

**Remark 4 (see [16])**

It is well known that for any *A* ∈ ℝ^*N*×*N*^, *A* ^*m*^ → 0 as *m* → *∞* if and only if *ρ*(*A*) ≤ 1. We note that if *A* is normal, then ||*A*|| = *ρ*(*A*) but when the matrix *A* is not normal the spectral radius gives no indication of the magnitude of the roundoff error for finite *M*. In this case a condition of the form *ρ*(*A*) ≤ 1 guarantees eventual decay of the errors, but does not control the intermediate growth of the errors. Then, it is easy to understand that *ρ*(*A*) ≤ 1 is a necessary condition for stability but not always sufficient.

## 5. The Fourier Stability of the Method

We analyze the stability of the difference scheme by a Fourier analysis. Let *U*_*j*_^*k*^ be the approximate solution and define *ρ*_*j*_^*k*^ = *u*_*j*_^*k*^ − *U*_*j*_^*k*^, *k* = 0,1,…, *N* − 1, *j* = 1,…, *M* − 1. Then, we write *ρ*_*j*_^*k*^ = *d*_*k*_ *e* ^*ijhβ*^ and obtain the following roundoff error equation for (9):

(24) 112[w0ρj−1k+1+∑r=1k(wr−wr−1)ρj−1k+1−r−wkρj−10]  +1012[w0ρjk+1+∑r=1k(wr−wr−1)ρjk+1−r−wkρj0]  +112[w0ρj+1k+1     +∑r=1k(wr−wr−1)ρj+1k+1−r−wkρj+10] =[ρj+1k+1−2ρjk+1+ρj−1k+12h2+ρj+1k−2ρjk+ρj−1k2h2],          0≤k≤N−1, 1≤j≤M−1,ρ0k=ρMk=0.

We now define the grid functions:

(25) $$\begin{array}{c} \rho^{k}\left(x\right)=\{\begin{array}{cc} \rho_{j}^{k}, & \text{when}\;\;x_{j-h/2}<x<x_{j+h/2}\\ 0, & \text{when}\;\;0\leq x\leq \frac{h}{2}\;\text{or}\;L-\frac{h}{2}<x\leq L, \end{array} \end{array}$$

and then *ρ* ^*k*^(*x*) can be expanded in a Fourier series as follows:

(26) $$\begin{array}{c} \rho^{k}\left(x\right)=\sum_{l=-\infty }^{\infty }d_{k}\left(l\right)e^{i2\pi lx/L},\;k=1,2,\ldots ,N, \end{array}$$

where *d*_*k*_(*l*) = 1/*L*∫_0_^*L*^ *ρ* ^*k*^(*x*)*e* ^−*i*2*πlx*/*L*^ and we introduce the following norm:

(27) $$\begin{array}{c} {||\rho^{k}||}_{2}={\left(\sum_{j=1}^{M-1}h{\left|\rho_{j}^{k}\right|}^{2}\right)}^{1/2}={\left[\int_{0}^{L}{\left|\rho^{k}(x)\right|}^{2}dx\right]}^{1/2}, \end{array}$$

and applying the Parseval equality ∫_0_^*L*^|*ρ* ^*k*^(*x*)|^2^ *dx* = ∑_*l*=−*∞*_^*∞*^|*d*_*k*_(*l*)|^2^, we obtain

(28) $$\begin{array}{c} {||\rho^{k}||}_{2}^{2}=\sum_{l=-\infty }^{\infty }{\left|d_{k}\left(l\right)\right|}^{2}. \end{array}$$

Based on the above analysis we can suppose that the solution of (24) has the following form *ρ*_*j*_^*k*^ = *d*_*k*_ *e* ^*ijhβ*^, where *β* = 2*πl*/*L* and *L* = 1. Substituting the above expression into (24), we obtain

(29) 112[w0dk+1ei(j−1)hβ  +∑r=1k(wr−wr−1)dk+1−rei(j−1)hβ−wkd0ei(j−1)hβ]  +1012[w0dk+1eijhβ     +∑r=1k(wr−wr−1)dk+1−reijhβ−wkd0eijhβ]  +112[w0dk+1ei(j+1)hβ     +∑r=1k(wr−wr−1)dk+1−rei(j+1)hβ        −wkd0ei(j+1)hβ] =[dk+1ei(j+1)hβ−2dk+1ei(j)hβ+dk+1ei(j−1)hβ2h2   +dkei(j+1)hβ−2dkei(j)hβ+dkei(j−1)hβ2h2],       0≤k≤N−1, 1≤j≤M−1,ρ0k=ρMk=0.

After simplifications, we write

(30) dk+1(w0(2cos⁡(βh)+10)12+1h2(1−cos⁡(βh))) =dk(1h2(cos⁡(βh)−1))  +(2cos⁡(βh)+10)12  ×[∑r=1k(wr−1−wr)dk+1−r]  +(wk(2cos⁡(βh)+10)12)d0.

**Theorem 5**

|*d*_*k*_| ≤ |*d*_0_| for *k* = 1,2,…, *N*, where *d*_*k*_ is the solution of (30).

**Proof**

We will use mathematical induction for the proof.

We start with *k* = 0, and then

(31) d1(w0(2cos⁡(βh)+10)12+1h2(1−cos⁡(βh))) =d0(1h2(cos⁡(βh)−1))  +(w0(2cos⁡(βh)+10)12)d0.

Then

(32) |d1|=|(w0(2cos⁡(βh)+10)/12)−(1/h2)(1−cos⁡(βh))(w0(2cos⁡⁡(βh)+10)/12)+(1/h2)(1−cos⁡(βh))| ×|d0|≤|d0|,

and therefore |*d*_1_| ≤ |*d*_0_|.

Now, assume that |*d*_*n*_| ≤ |*d*_0_|, *n* = 1,2,…, *k*. We need to prove that *n* = *k* + 1. Indeed,

(33) |dk+1|(w0(2cos⁡(βh)+10)12+1h2(1−cos⁡(βh))) ≤|dk||1h2(cos⁡(βh)−1)|  +|(2cos⁡⁡(βh)+10)12|∑r=1k|wr−1−wr|·|dk+1−r|  +|wk(2cos⁡⁡(βh)+10)12||d0|,|dk+1||w0(2cos⁡(βh)+10)12+1h2(1−cos⁡(βh))| ≤|d0||1h2(cos⁡(βh)−1)|  +|(2cos⁡(βh)+10)12|∑r=1k|wr−1−wr|·|d0|  +|wk(2cos⁡⁡(βh)+10)12||d0|,|dk+1|≤(|1h2(cos⁡⁡(βh)−1)|  +|(2cos⁡⁡(βh)+10)12(w0−wk)|  +|wk(2cos⁡⁡(βh)+10)12|) ×(|w012(2cos⁡⁡(βh)+10)+1h2(1−cos⁡⁡(βh))|)−1 ×|d0|,|dk+1|≤(−1h2(cos⁡(βh)−1)+(2cos⁡⁡(βh)+10)12(w0−wk)  +wk(2cos⁡⁡(βh)+10)12) ×(w012(2cos⁡⁡(βh)+10)+1h2(1−cos⁡(βh)))−1 ×|d0|,|dk+1|≤(w012(2cos⁡⁡(βh)+10)+1h2(1−cos⁡(βh))) ×(w012(2cos⁡(βh)+10)+1h2(1−cos⁡(βh)))−1 ×|d0|=|d0|.

**Theorem 6**

The finite difference scheme (9) is unconditionally stable.

**Proof**

Using the last theorem and Parseval equality, we obtain

(34) $$\begin{array}{c} {||\rho^{k}||}_{2}\leq {||\rho^{0}||}_{2},\;k=1,2,\ldots ,N, \end{array}$$

which means the proposed difference scheme is unconditionally stable.

## 6. Numerical Analysis

**Example 7**

One has

(35) ∂αu(t,x)∂tα=∂2u(t,x)∂x2+4!Γ(5−α)t7/2x5(1−x) +10(t4+1)x4−20(t4+1)(1−x)x3,           (0<x<1, 0<t<1),u(0,x)=0, 0≤x≤1,u(t,0)=0, u(t,1)=0, 0≤t≤1.

**Figure 1:**
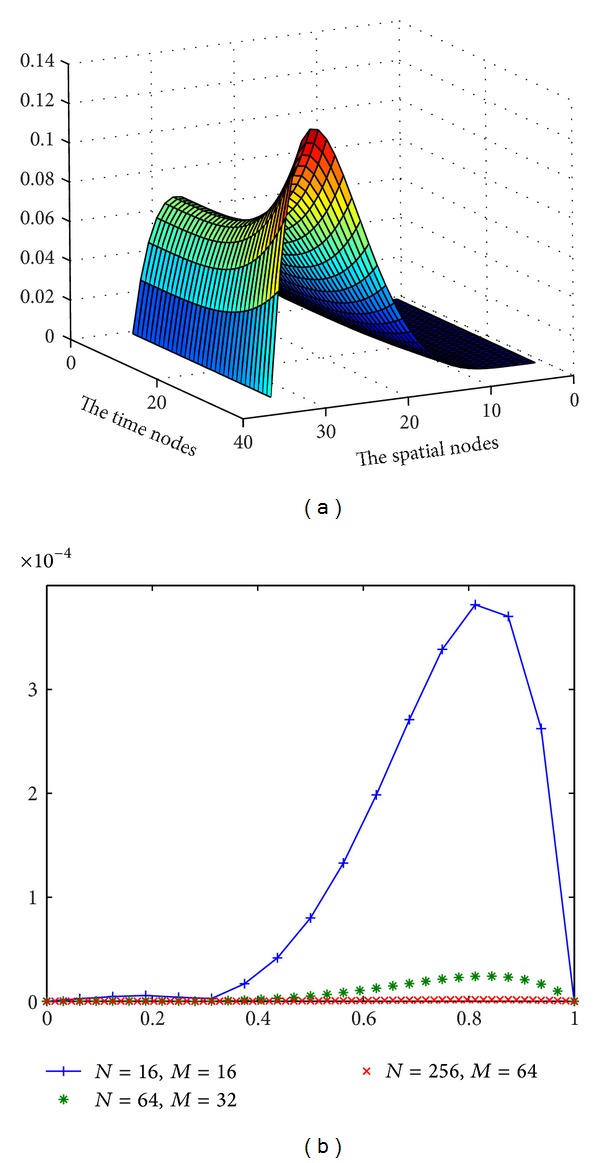
(a) The approximate solutions of Example 7 by the proposed method when *N* = 32, *M* = 32 and *α* = 0.5. (b) The errors for some values of *M* and *N* when *t* = 1 and *α* = 0.5.

Exact solution of this problem is *U*(*t*, *x*) = (*t* ^4^ + 1)*x* ^5^(1 − *x*). The solution by the proposed scheme is given in Figure 1. The errors when solving this problem are listed in Table 1 for various values of time and space nodes.

The errors in Table 1 are calculated by the following formula:

(36) $$\begin{array}{c} \underset{0\leq k\leq N}{\underset{0\leq n\leq M}{\max}}\left|u\left(t_{k},x_{n}\right)-U_{n}^{k}\right|. \end{array}$$

It can be concluded from Table 1 and Figure 1 above that when the time-step size is reduced by a factor of 1/4 and the spatial step size is reduced by a factor of 1/2, then the error decreases by about 1/16. The numerical results support the claim about the order of the convergence.

## 7. Conclusion

In this work, the compact difference scheme was successfully applied to solve the time fractional heat equations. The second order approximation for the Riemann-Liouville fractional derivative is equipped with the higher order compact difference schemes. The Fourier analysis and the spectral stability method are used to show that the proposed scheme is unconditionally stable. Numerical results are in good agreement with the theoretical results.

## Figures and Tables

**Table 1:** Error table for Example 7.

*M*	*N*	*α* = 0.3	*α* = 0.5	*α* = 0.8
		Error	Error	Error
8	8	0.0014442	0.0014297	0.0014076
16	32	0.0000910	0.0000908	0.0000898
32	128	0.0000058	0.0000058	0.0000058
